# Innovative targets of the lncRNA-miR-mRNA network in response to low-dose aspirin in breast cancer patients

**DOI:** 10.1038/s41598-022-16398-7

**Published:** 2022-07-14

**Authors:** Sadaf Alipour, Solmaz Khalighfard, Vahid Khori, Taghi Amiriani, Mahboubeh Tajaldini, Mohammad Dehghan, Somayeh Sadani, Ramesh Omranipour, Gelareh Vahabzadeh, Bita Eslami, Ali Mohammad Alizadeh

**Affiliations:** 1 Breast Diseases Research Center, Cancer Institute, Tehran University of Medical Sciences, Tehran, Iran; 2 Department of Surgery, Arash Women’s Hospital, Tehran University of Medical Sciences, Tehran, Iran; 3 Department of Biology, Science and Research Branch, Islamic Azad University, Tehran, Iran; 4 Division of Gastroenterology Hepatology and Nutrition, Department of Medicine, College of Medicine, University of Florida, Gainesville, FL USA; 5 Ischemic Disorders Research Center, Golestan University of Medical Sciences, Gorgan, Iran; 6 Department of Pharmacology, School of Medicine, Iran University of Medical Science, Tehran, Iran; 7 Cancer Research Center, Cancer Institute, Tehran University of Medical Sciences, Tehran, Iran

**Keywords:** Biological techniques, Cancer, Cell biology, Chemical biology, Computational biology and bioinformatics, Developmental biology, Genetics, Immunology, Molecular biology, Biomarkers, Diseases, Molecular medicine, Oncology

## Abstract

This study aimed to investigate innovative targets in breast cancer patients by considering the interaction of the lncRNA-miR-mRNA network in response to low-dose aspirin. The candidate miRs were first taken from the GEO and TCGA databases. Then, the candidate network was constructed using the high-throughput sequencing data. The expression levels of candidate targets were finally measured using Real-Time PCR in luminal A breast cancer patients undergoing aspirin (80 mg daily for three months) and non-aspirin groups during chemotherapy after surgery. The expression levels of TGFβ, IL-17, IFNγ, and IL-β proteins were measured using the ELISA technique. 5 lncRNAs, 12 miRs, and 10 genes were obtained in the bioinformatic phase. A significant expression increase of the candidate tumor suppressor lncRNAs, miRs, and genes and a substantial expression decrease of the candidate onco-lncRNAs, oncomiRs, and oncogenes were achieved after the aspirin consumption. Unlike the non-aspirin group, the expression levels of TGFβ, IL-17, IFNγ, and IL-β proteins were significantly decreased following aspirin consumption. The Kaplan–Meier analysis indicated a longer overall survival rate in the patients after aspirin consumption. Our results showed that the lncRNA-miR-mRNA network might be a significant target for aspirin; their expression changes may be a new strategy with potential efficacy for cancer therapy or prevention.

## Introduction

Inflammation predisposes to cancer development and promotes all stages of tumorigenesis. Its inhibition can hinder tumor growth and progress, increase the chances of early detection, and shed light on how metastatic seeds outgrow once distantly established^1^. Accordingly, anti-inflammatory drugs could reduce the risk of cancer and cancer-related deaths. The epidemiologic studies disclosed the inverse correlation between non-steroidal anti-inflammatory drugs and breast cancer incidences. Recent meta-analyses of observational studies have revealed that aspirin (acetylsalicylic acid; aspirin) could reduce the risk of breast cancer due to its anti-inflammatory effects, mainly in the hormone receptor-positive breast cancer subtype^2,3^. Early investigations have already established that aspirin could inhibit cyclooxygenase 2 (COX2) activity and reduce prostaglandin E2 (PGE2) production, both overexpressed in breast cancer. In this setting, aspirin may act, at least in part, by suppressing aberrant nuclear factor-κB (NF-κB) signaling that can promote tumor cell survival, proliferation, migration, invasion, angiogenesis, and resistance to therapy. Other anti-cancer mechanisms for aspirin have included inhibiting cyclooxygenase^4^, activating AMPK, mTOR, STAT3, and NF-κB pathways^5^, decreasing reactive oxygen species (ROS)^6^, inducing autophagy^7^, and changing tumor microenvironment^8^. Moreover, aspirin could meditate its anti-cancerous properties by changing the expression of non-coding RNAs, such as microRNAs (miRs)^9^. Accordingly, McDonald et al. (2018) showed that aspirin could alter the expression of miRs in endometrial cancer cells in a dose-dependent manner^9^. Other studies demonstrated the increased expression of miR-340-5p and miR-137 by inhibiting cancer cells' proliferation and decreasing cyclin D1 and miR-7-5p expression due to aspirin consumption^10,11^.

Furthermore, the anti-proliferative effects of aspirin could also be through the regulation of the long non-coding RNAs (lncRNAs), inducing OLA1P2 expression through FOXD3 upregulation^12^. Wang et al. (2019) reported that aspirin could reduce the P4AH2 expression via let-7g up-regulation, restraining the axis of NF-kB/P4HA2 and LMCD1-AS1/let-7g/P4HA2^13^. Therefore, lncRNAs can act by miR response elements or binding sites to bind to their target mRNAs, forming a ceRNA network^14^. For instance, the pseudogene PTENP1 lncRNA has the miR sponge capacity to regulate the PTEN gene^15^. In addition, H19 is a cytoplasmic lncRNA that has been shown to bind preference to let-7, promoting the cancer stem cells by making a reciprocal negative feedback loop with let-7 target^14,16^. In this setting, Fan et al. (2018) constructed a lncRNA-miR-mRNA network and showed four lncRNAs, which have prognostic values in breast cancer patients^17^. This issue necessitates in-depth analyses of these networks in breast cancer. Therefore, we aimed for a randomized trial with a systematic approach that introduced innovative targets in breast cancer by considering the interaction of the lncRNA-miR-mRNA network in response to low-dose aspirin.

## Results

### Patients and tumor characteristics

Figure 11 demonstrates the flow chart of the patients. Patients in the intervention groups, five in the Aspirin group and four in the non-aspirin group, were withdrawn from the study because the physicians decided only to prescribe endocrine therapy for their systemic adjuvant treatment. Three patients in the Aspirin group and one in the non-aspirin group did not follow the research. Therefore, 40 patients, including 20 in the Aspirin group and 20 in the non-aspirin group, were entered into the data analysis. In addition, the mean age of the patients in the Aspirin and non-aspirin groups and healthy individuals was 57 ± 11, 53 ± 8, and 49 ± 7, respectively. The mean tumor size was 1.6 ± 0.5 mm in the Aspirin group and 1.8 ± 0.3 in the non-aspirin group. According to the inclusion criteria, all tumors were positive for hormone receptors, negative for Her2, and their Ki-67 was less than 15.

### Identification of differentially expressed genes (DEGs)

**Figure 1:**
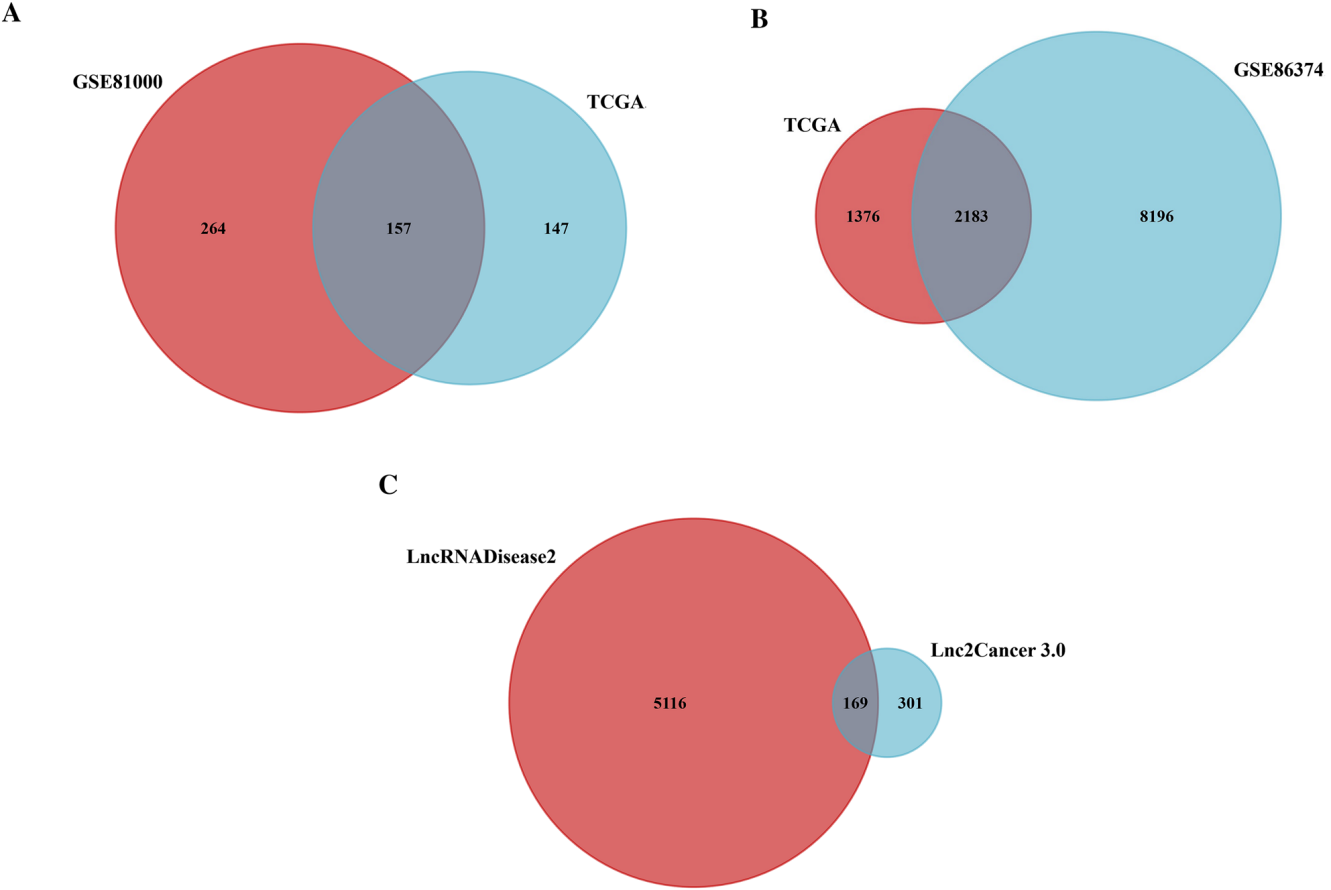
A Venn diagram of the differently expressed lncRNAs, miRs, and mRNAs between GEO and TCGA datasets. Allocation of (**A**) the 157 differently expressed miRs (37 up-regulation and 120 down-regulation), (**B**) 2183 differently expressed mRNAs (996 up-regulation and 1187 down-regulation), and (**C**) 169 differently expressed lncRNAs (102 up-regulation and 67 down-regulation) was found between the datasets in the present study.

FunRich_3.1.3 software made a Venn diagram and extracted the candidate datasets (Fig. 1). A total of 157 miRs (37 up-regulation and 120 down-regulation) (Fig. 1A), 2183 mRNAs (996 up-regulation and 1187 down-regulation) (Fig. 1B), and 169 lncRNAs (102 up-regulation and 67 down-regulation) (Fig. 1C) were obtained from the candidate datasets.The top up-regulated miRs included miR-21, miR-10b, miR-155, miR-20a, miR-20b, miR-141, and miR-200a. The top down-regulated miRs were miR-145, miR-224, miR-125a, and miR-205 (Table 1). Targetscan, miRmap, and mirwalk2 software identified the target genes, including *TGFβR2, PIK3CD, AKT3, ERBB2 (HER2), MYC, NOTCH1, IGF1, PTEN, FOXO3,* and *SOCS5* (Tables 2, 3, and 4). Besides, the LncRNA2target, LncRNADisease v2.0, Lnc2cancer v3.0, and TANRIC datasets identified the candidate lncRNAs, including *MALAT1, HOTAIR, XIST, GAS5,* and *ZFAS1* (Table 5).

**Table 1:** The candidate miRs in breast cancer patients.

miRs	adj.P.Val	logFC
**Up-regulated**		
miR-21	5.51E−06	− 2.84086
miR-10b	0.000847401	− 2.173494
miR-155	0.000444277	− 3.20482
miR-17	0.000878375	− 2.02905
miR-141	0.000338	− 1.78044
miR-200a	0.000307	− 1.80332
miR-20a	0.000946717	− 2.04178
miR-20b	0.000948413	− 1.02111
**Down-regulated**		
miR-145	0.035118	2.771778
miR-224	0.009643	2.032727
miR-125a	0.0046119	2.3917
miR-205	0.00019275	2.959589

**Table 2:** Interaction analysis between selected miRs and target genes in breast cancer patients.

miRs	Target genes
miR-21	AKT2, APC,APPL1,BCL2,CCND1,CYCS,EGFR, IGFIR, MSH2,MSH6, MYC,PI3KR1, IGFB1, IGFB2, IGFBR2, PTEN
miR-20a	ACVRIB, BCL2, CCND1, CYCS, FZD9, MAPK1, MAPK9, MSH3, MYC, SMAD4, TCF7L2, TGFβR2, TP53
miR-20b	ACVRIB, CCND1, CYCS, FZD9, FZD4, MAPK1, MAPK9, MSH3, SMAD4, TCF7L2, TGFβR2
miR-125a	E2F2, HEYL, PIK3R3, JUN, GADD45A, SHC1, AKT3, NCOA1, CTNNB1, WNT5A, GSK3B, PIK3CB, DVL3, KIT, LEF1, FGF2, PIK3R1, POLK, TCF7, FGF1, FGF18, BAK1, ESR1, CDK6, WNT16, BRAF, FZD3, MYC, FRAT2, FGF23, IGF1, WNT10B, IGF1R, TP53, BRCA1, WNT3, SHC2, APC2, AKT2, BAX, NCOA3, MAPK1, ERBB2
miR-141	NOTCH2, SOS1, WNT5A, FGF5, PIK3R1, WNT8A, FGF18, E2F3, HEY2, ESR1, FRAT1, DDB2, CDK4, IGF1R, WNT9B, RPS6KB1, AP1, HMGA1, STAT6, JAK1
miR-145	WNT4, E2F2, HEYL, JUN, WNT9A, AKT3, RAF1, WNT5A, GSK3B, PIK3CB, PIK3CA, PIK3R1, FGF1, CDK6, WNT16, BRAF, MYC, PTEN, FRAT1, FRAT2, SP1, FGF9, ESR2, IGF1R, GADD45B, JAG1, WNT7B,
miR-155	FGF1, BRAF, SP1, IGF1R, FZD2, WNT3, WNT9B, E2F1
miR-17	PIK3R3, WNT2B, SOS1, WNT5A, GSK3B, KIT, FGF5, FGF2, PIK3R1, POLK, APC, FGF1, E2F3, NOTCH4, BAK1, ESR1, CDK6, WNT16, BRAF, FZD3, FGFR1, MYC, SHC3, DDB2, WNT10B, SP1, TNFSF11, RB1, JAG2, FGF7, MAP2K1, MAPK3, TP53, BRCA1, WNT9B, RPS6KB1, AXIN2, PIK3R2, AKT2, NCOA3, WNT7B, ARAF
miR-200a	NRAS, NOTCH2, LEF1, PIK3R1, CDK6, FGFR1, LRP6, KRAS, WNT10B, IGF1, FGF7, IGF1R, RPS6KB1, GADD45B, JAG1, E2F1, ZEB
miR-205	HES5, AKT3, RAF1, PIK3CB, KIT, FGF2, PIK3R1, POLK, APC, FGF1, CSNK1A1, CDK6, WNT2, WNT16, BRAF, FZD3, FGFR1, FRAT2, TCF7L2, LRP6, KRAS, SP1, FGF9, TNFSF11, DLL4, TP53, FZD2, WNT3, AKT2, E2F1, VEGFA
miR-224	WNT4, E2F2, PIK3R3, WNT9A, AKT3, RAF1, PIK3CB, FGF2, PIK3R1, FGF1,CDK6, FZD3,FGF8, TCF7L2, WNT5B, KRAS, SP1, CDK4, FZD10, RB1, ESR2, BRCA1, BAX, NCOA3, MAPK1, ETFRF1, AP1, SOCS7, SOCS5, HOXD10
miR-10b	GABRB1, SESN3, RB1CC1, ZDHHC21, TFAP2A, NUFIP2, LHFPL4, GATAD2A, CHD6, ONECUT2, ZDHHC18, MAP3K2, AGO3, SHISA7, UNC5B, DLEC1, DLG5, PAPOLA, FBXO28, HDAC4, INO80D, KIAA1549, L3MBTL3, ZBTB43, USF2, BCL2L2, MIEF1, IFFO2, AAK1, INHBB, GCLM, RBM27, TRIM66, FOSL2, ARIH2, MTF1, CCNK, NFIX, TBC1D22B, LPHN1, HOXA1, SNX12, FXR2, CLASP2, SH3D19, NR2C2, ELAVL2, RYBP, PCDH10, RNF156, ESRRG, HES5, SHC1, NCOA1, WNT5A, GSK3B, DVL3, PIK3R1, WNT8A, E2F3, EGFR, WNT2, FZD3, FGF23, WNT8B, WNT1, IGF1, ESR2

**Table 3:** The predicted candidate genes in breast cancer patients.

Up-regulated	AKT3, FGF5, PIK3R1, FGF1, NOTCH4, AKT2, PIK3R3, WNT2B, WNT5A, FGF2, WNT16, BRAF, MYC, WNT10B, PIK3R2, FGF7, MAP2K1, MAPK3, WNT9B, WNT7B, ARAF, FGFR1, NOTCH2, WNT8A, FGF18, IGF1R, STAT6, JAK1, WNT9A, RAF1, PIK3CA, AKT1, IGF1, FGF9, MAPK1, WNT8B, EGFR, KRAS, NRAS, VEGFA, PIK3CB, WNT2, WNT3, WNT1, FZD3, FZD1, FZD2, FZD9, SHC3, LRP6, RPS6KB1, KIT, CDK6, JAG2, FLT4, LEF1, NCOA3, HEY2, CDK4, HMGA1, HES5, GADD45B, DLL4, TGFβR2, TWIST, BCL2, CCND1, IGFIR, PI3KR1, IGFB1, IGFB2, IGFBR2, FZD9, MAPK9, SMAD4, FZD4, MAPK9, WNT4, FGF23, FZD10, FGF8, WNT5B, MAP3K2, POLK, RPS6KB2, PIK3CD, NCOA1, TFAP2A, APPL1, JAG1, JUN, SHC1, SHC2, CTNNB1, DVL3, DVL1, ERBB2, CDKN1A, SOCS7, SOCS5, SESN3, BCL2L2, GABRB1, NUFIP2
Down-regulated	E2F2, APC, E2F1, E2F3, BRCA1, BAK1, TP53, SOS1, SP1, SOS2, ESR1, ESR2, FRAT2, FRAT1, TNFSF11, RB1, AXIN2, GSK3B, POLK, DDB2,AP1, TCF7, TCF7L2, CDKN1A, ZEB, CSNK1A1, HOXD11, PTEN, FOXO3, PDCD4, BRCA1, APC2, BAX, SP1, HOXD10, FRAT2, DDB2, ESRRG, CYCS, MSH2,MSH6, MSH3, ACVR1B, KIT, HEYL, GADD45A, CSNK1A1, CSNK1A1L, TNFSF11, SCH3, AXIN2, ZDHHC21

**Table 4:** The list of selected genes involved in breast cancer patients.

Genes	Target miRs
NOTCH1	miR-141, miR-17, miR-200a
TGFβR2	miR-17
MYC	miR-21, miR-20a, miR-125a, miR-145
PIK3CD	miR-125a, miR-145, miR-224
AKT3	miR-21, miR-125a, miR-145, miR-224
ERBB2	miR-125a
IGF1	miR-21, miR-155, miR-10b, miR-20a, miR-20b, miR-224, miR-145
SOCS5	miR-106a, miR-141, miR-155, miR-200a, miR-342, miR-21, miR-20a, miR-20b, miR-224, miR-205
PTEN	miR-21, miR-145
FOXO3	miR-224, miR-155, miR-125a, miR-10b, miR-21

**Table 5:** Interaction analysis between selected miRs and lncRNAs in breast cancer patients.

LncRNAs	Target miRNAs
MALAT1	miR-10b, miR-125a, miR-141, miR-145, miR-155, miR-17,miR-200a, miR-205, miR-20a, miR-20b, miR-21, and miR-224
GAS5	miR-10b, miR-141,miR-155, miR-205, miR-20a, miR-20b, miR-21, and miR-224
XIST	miR-10b, miR-125a, miR-141, miR-145, miR-155, miR-17, miR-200a, miR-205, miR-20a, miR-20b, miR-21, and miR-224
HOTAIR	miR-10b, miR-145, miR-17, miR-205, miR-20a, miR-20b, and miR-21
ZFAS1	miR-10b, miR-145, miR-17, and miR-21

### Enrichment analysis of DEGs

**Figure 2:**
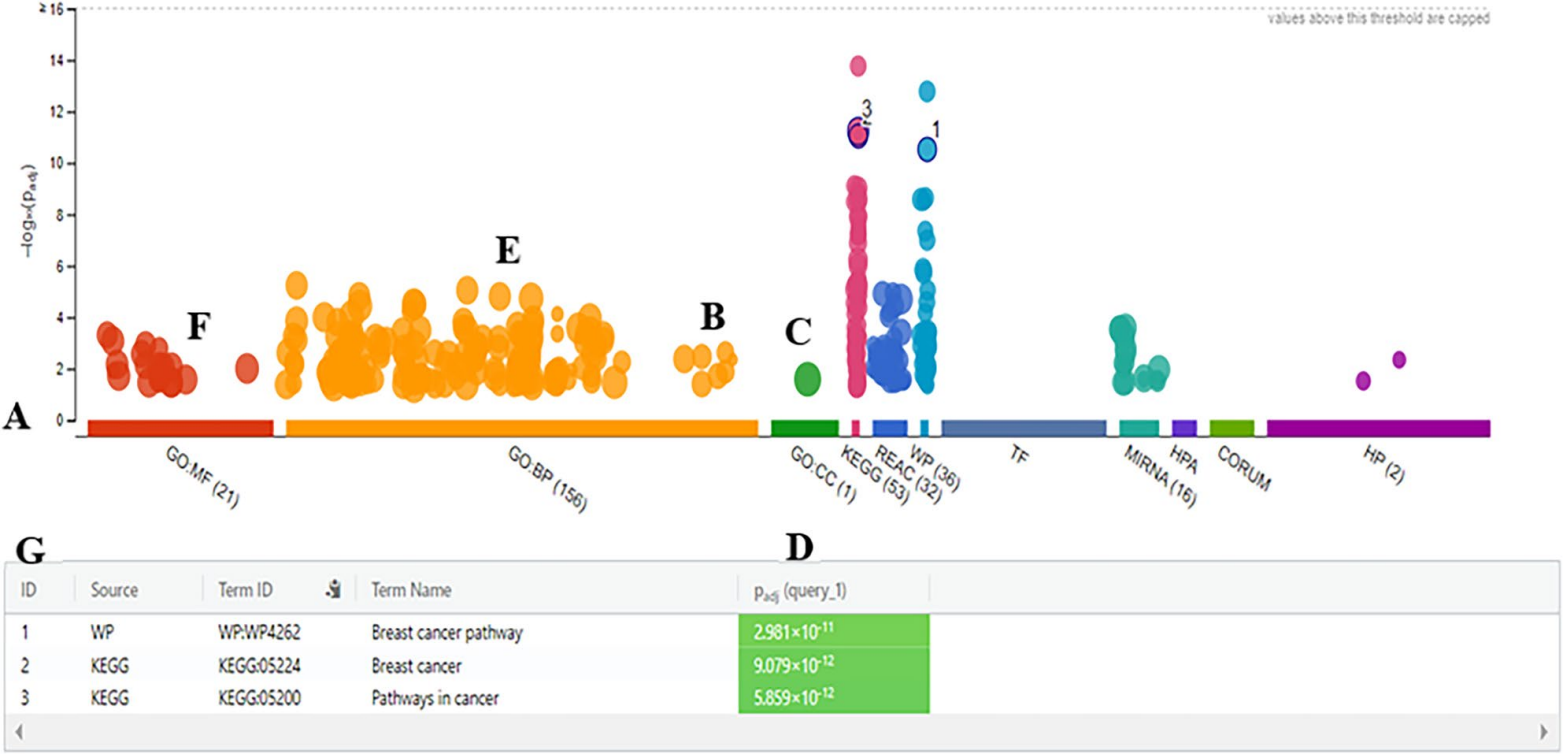
Functional enrichment by the g: Profiler software. (**A**) the X-axis shows the functional terms grouped and color-coded by the data source. (**B**, **C**, **F**) the position of terms in the plots fixed and terms from the same branch of Gene Ontology. (**D**) p-values in the table outputs are color-coded from yellow (insignificant) to blue (highly significant). (**E**) in a multi-query case, the same term is highlighted on other plots. (**G**) a click allows for pinning the circles to the plot with a numeric ID that creates a more detailed result in the table below the image.

Gene Ontology (GO) analysis was performed by FunRich to evaluate the biological functions of DEGs. The pathways were specifically enriched by DEGs, including receptor binding, cell cycle, proliferation, transcription factor activity, serine/threonine kinase activity, growth factor activity, DNA repair protein, EGF receptor, glypican, ErbB, and Rap1 (Table 6). All candidate miRs could regulate the signaling pathways, including Wnt, PI3K-AKT, EGF, NOTCH, JAK/STAT, and apoptosis. The most modified routes were the PI3K/AKT and WNT pathways. Thus, candidate miRs could target the significant genes involved in these pathways (Table 6). As shown in Fig. 2, functional enrichment of DEG genes was also analyzed using g: Profiler software.

**Table 6:** Integrative pathway enrichment analysis for DEGs.

Term_ID	Term_name	adj_P_value
**GO: BP**		
positive regulation of protein phosphorylation	GO:0001934	5.773 × 10–6
positive regulation of phosphate metabolic process	GO:0045937	1.554 × 10–5
positive regulation of the cellular metabolic process	GO:0031325	3.100 × 10–5
mammary gland development	GO:0030879	5.701 × 10–5
protein phosphorylation	GO:0006468	1.006 × 10–4
cellular response to organic substance	GO:0071310	1.155 × 10–4
regulation of transferase activity	GO:0051338	1.234 × 10–4
regulation of protein phosphorylation	GO:0001932	1.419 × 10–4
positive regulation of macromolecule metabolic process	GO:0010604	3.616 × 10–5
growth	GO:0040007	1.536 × 10–4
positive regulation of transferase activity	GO:0051347	1.882 × 10–4
cell development	GO:0048468	2.377 × 10–4
regulation of growth	GO:0040008	2.632 × 10–4
regulation of molecular function	GO:0065009	2.644 × 10–4
cellular response to growth factor stimulus	GO:0071363	3.138 × 10–4
positive regulation of cellular protein metabolic process	GO:0032270	3.251 × 10–4
regulation of phosphorylation	GO:0042325	3.269 × 10–4
response to growth factor	GO:0070848	4.126 × 10–4
embryonic organ development	GO:0048568	4.374 × 10–4
regulation of signal transduction	GO:0009966	5.169 × 10–4
circulatory system development	GO:0072359	5.191 × 10–4
positive regulation of protein metabolic process	GO:0051247	5.589 × 10–4
blood vessel development	GO:0001568	5.608 × 10–4
response to endogenous stimulus	GO:0009719	5.759 × 10–4
regulation of protein kinase activity	GO:0045859	7.087 × 10–4
phosphorylation	GO:0016310	7.136 × 10–4
positive regulation of molecular function	GO:0044093	7.358 × 10–4
vasculature development	GO:0001944	7.583 × 10–4
cardiovascular system development	GO:0072358	8.177 × 10–4
positive regulation of MAP kinase activity	GO:0043406	1.119 × 10–3
regulation of protein modification process	GO:0031399	1.187 × 10–3
regulation of kinase activity	GO:0043549	1.354 × 10–3
positive regulation of protein kinase activity	GO:0045860	1.534 × 10–3
regulation of cell communication	GO:0010646	1.722 × 10–3
positive regulation of MAPK cascade	GO:0043410	1.841 × 10–3
positive regulation of the biological process	GO:0048518	1.946 × 10–3
regulation of signaling	GO:0023051	1.947 × 10–3
positive regulation of catalytic activity	GO:0043085	2.219 × 10–3
MAPK cascade	GO:0000165	2.405 × 10–3
cell population proliferation	GO:0008283	2.426 × 10–3
positive regulation of kinase activity	GO:0033674	2.432 × 10–3
**GO: MF**		
protein kinase activity	GO:0004672	4.844 × 10–4
signaling receptor binding	GO:0005102	8.392 × 10–4
phosphatase binding	GO:0019902	1.702 × 10–3
kinase activity	GO:0016301	2.682 × 10–3
ATP binding	GO:0005524	6.829 × 10–3
transferase activity, transferring phosphorus-containing groups	GO:0016772	7.224 × 10–3
adenyl ribonucleotide binding	GO:0032559	8.755 × 10–3
enzyme binding	GO:0019899	8.948 × 10–3
protein kinase binding	GO:0019901	1.443 × 10–2
drug binding	GO:0008144	1.906 × 10–2
mitogen-activated protein kinase kinase kinase binding	GO:0031435	1.917 × 10–2
kinase binding	GO:0019900	2.499 × 10–2
purine ribonucleoside triphosphate binding	GO:0035639	2.641 × 10–2
**GO: CC**		
cytoplasmic part	GO:0044444	2.581 × 10–2
**KEGG**		
Breast cancer	KEGG:05224	9.079 × 10–12
Pathways in cancer	KEGG:05200	5.859 × 10–12
EGFR tyrosine kinase inhibitor resistance	KEGG:01521	7.808 × 10–10
Endocrine resistance	KEGG:01522	3.173 × 10–9
Proteoglycans in cancer	KEGG:05205	1.214 × 10–8
Endometrial cancer	KEGG:05213	1.220 × 10–8
Central carbon metabolism in cancer	KEGG:05230	3.240 × 10–6
Cellular senescence	KEGG:04218	5.472 × 10–6
MicroRNAs in cancer	KEGG:05206	6.265 × 10–6
MAPK signaling pathway	KEGG:04010	8.334 × 10–6
PI3K-Akt signaling pathway	KEGG:04151	2.790 × 10–5
FoxO signaling pathway	KEGG:04068	7.248 × 10–5
ErbB signaling pathway	KEGG:04012	4.710 × 10–4
PD-L1 expression and PD-1 checkpoint pathway in cancer	KEGG:05235	5.932 × 10–4
Focal adhesion	KEGG:04510	6.213 × 10–4
HIF-1 signaling pathway	KEGG:04066	1.327 × 10–3
Sphingolipid signaling pathway	KEGG:04071	1.876 × 10–3
Osteoclast differentiation	KEGG:04380	2.277 × 10–3
Relaxin signaling pathway	KEGG:04926	2.499 × 10–3
mTOR signaling pathway	KEGG:04150	4.896 × 10–3
Adherens junction	KEGG:04520	1.093 × 10–2
Progesterone-mediated oocyte maturation	KEGG:04914	2.750 × 10–2
Th17 cell differentiation	KEGG:04659	3.374 × 10–2
**REACTUM**		
Negative regulation of the PI3K/AKT network	REAC: R-HSA-199418	1.167 × 10–5
Diseases of signal transduction	REAC: R-HSA-5663202	1.221 × 10–5
Signal Transduction	REAC: R-HSA-162582	1.980 × 10–5
PIP3 activates AKT signaling	REAC: R-HSA-1257604	3.592 × 10–5
Intrinsic Pathway for Apoptosis	REAC: R-HSA-109606	6.437 × 10–5
Intracellular signaling by second messengers	REAC: R-HSA-9006925	8.534 × 10–5
Signaling by Interleukins	REAC: R-HSA-449147	3.810 × 10–4
PI3K/AKT Signaling in Cancer	REAC: R-HSA-2219528	6.145 × 10–4
Activation of BH3-only proteins	REAC: R-HSA-114452	1.241 × 10–3
FLT3 Signaling	REAC: R-HSA-9607240	1.396 × 10–3
Oncogene Induced Senescence	REAC: R-HSA-2559585	1.665 × 10–3
Other interleukin signaling	REAC: R-HSA-449836	2.144 × 10–3
Generic Transcription Pathway	REAC: R-HSA-212436	2.415 × 10–3
Cytokine Signaling in Immune system	REAC: R-HSA-1280215	2.836 × 10–3
Activation of NOXA and translocation to mitochondria	REAC: R-HSA-111448	3.279 × 10–3
PTEN Regulation	REAC: R-HSA-6807070	3.433 × 10–3
RNA Polymerase II Transcription	REAC: R-HSA-73857	4.995 × 10–3
Apoptosis	REAC: R-HSA-109581	8.618 × 10–3
MAPK1 (ERK2) activation	REAC: R-HSA-112411	9.164 × 10–3
Programmed Cell Death	REAC: R-HSA-5357801	9.220 × 10–3
Gene expression (Transcription)	REAC: R-HSA-74160	1.032 × 10–2
Activation of PUMA and translocation to mitochondria	REAC: R-HSA-139915	1.177 × 10–2
Disease	REAC: R-HSA-1643685	1.294 × 10–2
Extra-nuclear estrogen signaling	REAC: R-HSA-9009391	1.927 × 10–2
Downregulation of ERBB2:ERBB3 signaling	REAC: R-HSA-1358803	2.155 × 10–2
TP53 Regulates Metabolic Genes	REAC: R-HSA-5628897	2.619 × 10–2
RAF/MAP kinase cascade	REAC: R-HSA-5673001	2.758 × 10–2
TP53 Regulates Transcription of Genes Involved in G1 Cell Cycle Arrest	REAC: R-HSA-6804116	2.967 × 10–2
Regulation of TP53 Activity through Association with Co-factors	REAC: R-HSA-6804759	2.967 × 10–2
MAPK1/MAPK3 signaling	REAC: R-HSA-5684996	2.995 × 10–2
PI5P, PP2A, and IER3 Regulate PI3K/AKT Signaling	REAC: R-HSA-6811558	3.343 × 10–2
TFAP2 (AP-2) family regulates transcription of growth factors and their receptors	REAC: R-HSA-8866910	3.421 × 10–2
**WP**		
Breast cancer pathway	WP: WP4262	2.981 × 10–11
Integrated Breast Cancer Pathway	WP: WP1984	2.633 × 10–9
Endometrial cancer	WP: WP4155	4.450 × 10–8
DNA Damage Response (only ATM dependent)	WP: WP710	1.351 × 10–6
ErbB Signaling Pathway	WP: WP673	2.591 × 10–5
Integrated Cancer Pathway	WP: WP1971	6.752 × 10–5
EGF/EGFR Signaling Pathway	WP: WP437	4.824 × 10–4
Leptin signaling pathway	WP: WP2034	6.182 × 10–4
PI3K-Akt Signaling Pathway	WP: WP4172	1.074 × 10–3
Focal Adhesion	WP: WP306	1.288 × 10–3
Senescence and Autophagy in Cancer	WP: WP615	2.411 × 10–3
TCA Cycle Nutrient Utilization and Invasiveness of Ovarian Cancer	WP: WP2868	2.425 × 10–3
MAPK Signaling Pathway	WP: WP382	3.757 × 10–3
Focal Adhesion-PI3K-Akt-mTOR-signaling pathway	WP: WP3932	9.986 × 10–3
RAC1/PAK1/p38/MMP2 Pathway	WP: WP3303	1.751 × 10–2

### Protein–protein interaction (PPI) network analysis of DEGs

**Figure 3:**
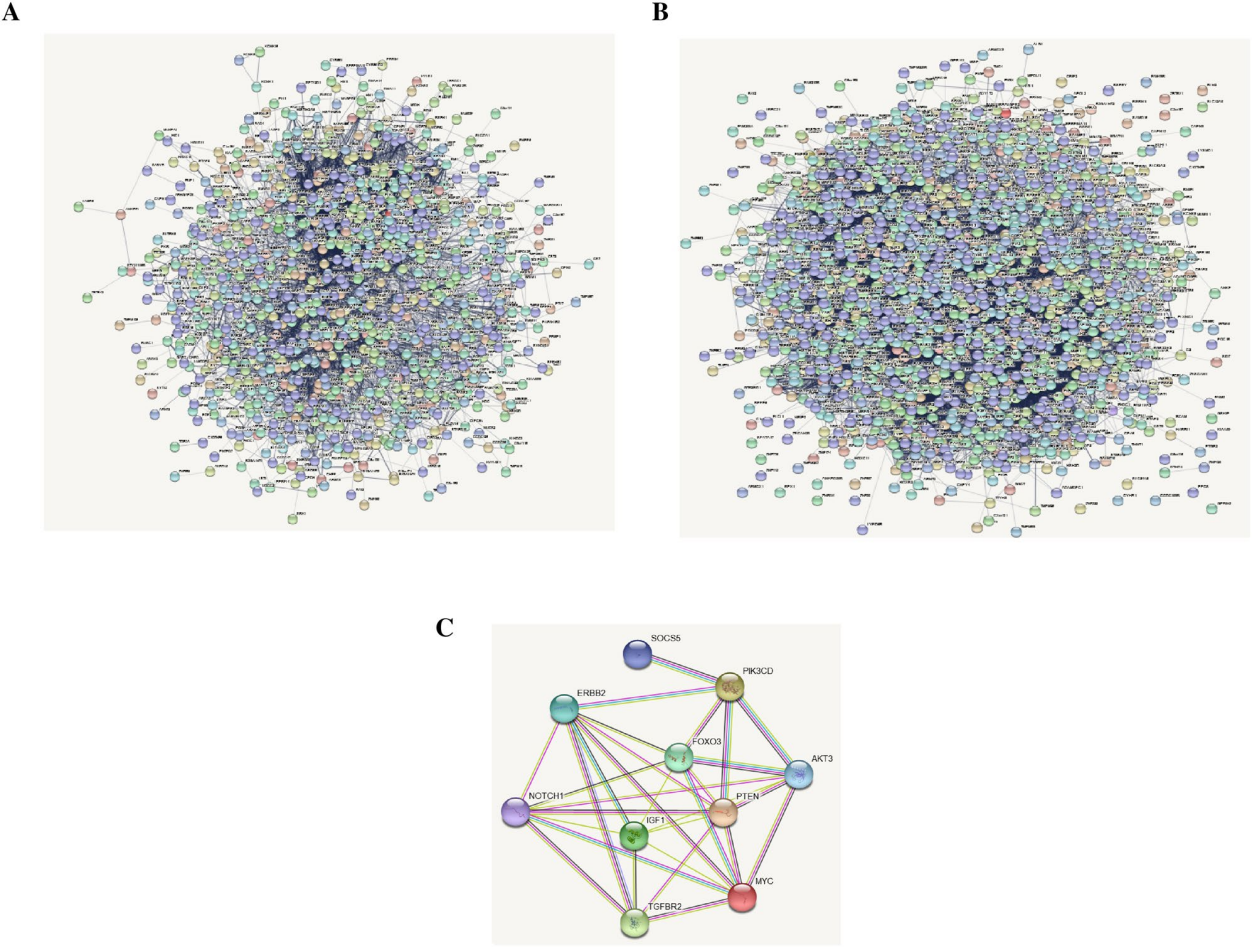
Protein–protein interaction (PPI) network. PPI network was constructed with the DEGs from the GEO and TCGA datasets. (**A**,**B**) The significant interactions were identified from the PPI network using the STRING database with a score of ≥ 7. (**A**) the interaction of up-regulated genes, (**B**) the interaction of down-regulated genes, and (**C)** the interaction between up-and-downregulation genes.

PPI analysis of the 2183 DEGs was performed in the FunRich software (score ≥ 7). *TGFβR2, PIK3CD, AKT3, ERBB2, MYC, NOTCH1,* and *IGF1* were hub nodes with higher node degrees in up-regulated genes (Fig. 3A). *PTEN, FOXO3,* and *SOCS5* were hub node degrees in down-regulated genes (Fig. 3B). As a result, *TGFβR2, PIK3CD, AKT3, ERBB2, MYC, NOTCH1, IGF1, PTEN, FOXO3,* and *SOCS5* were selected as hub genes for further analysis to the high degree of connectivity (Fig. 3C).

### Construction of lncRNA-miR-mRNA network

**Figure 4:**
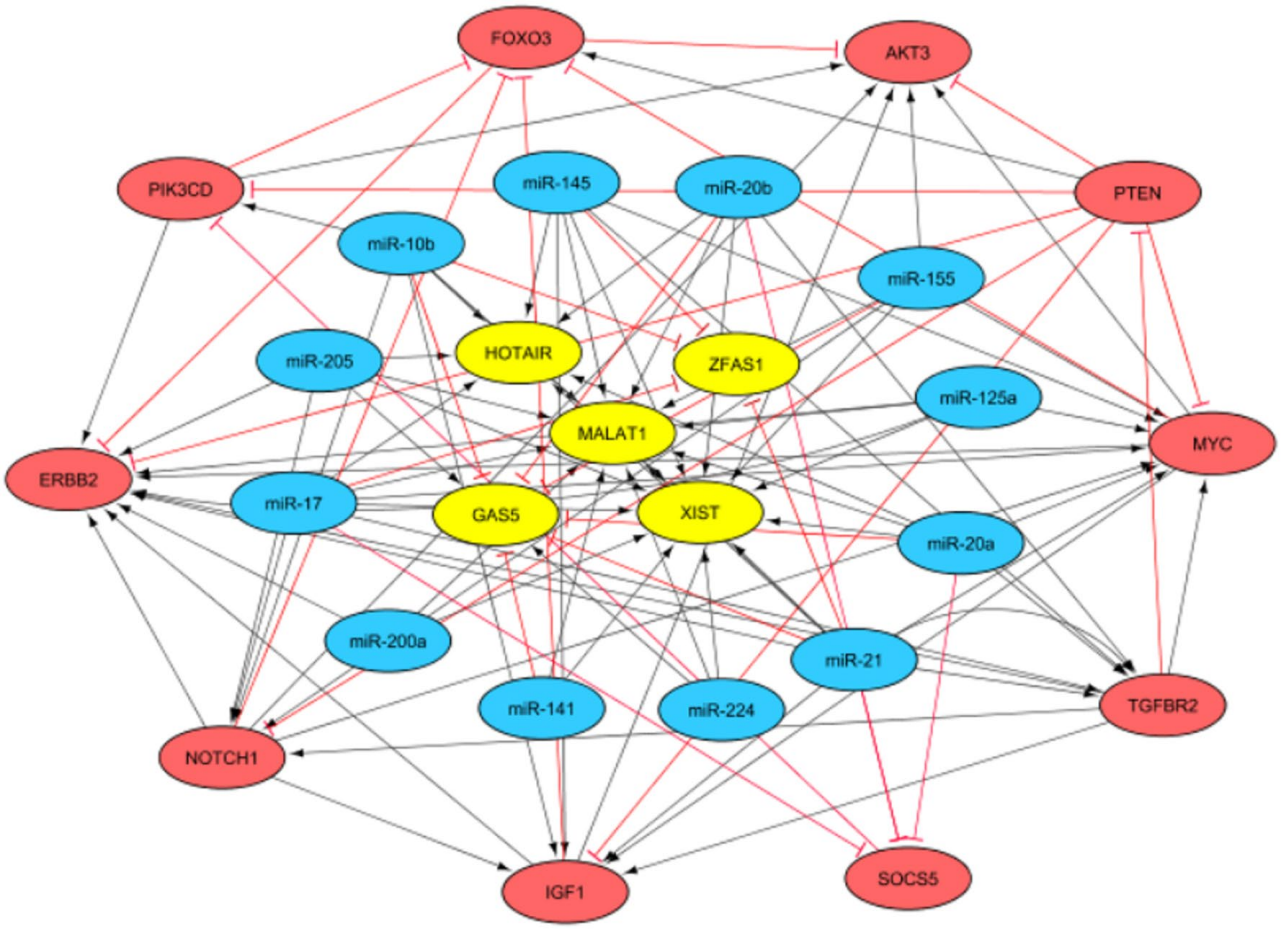
ceRNA regulatory network of lncRNAs, miRs, and mRNAs in breast cancer samples. The network includes 27 nodes and 103 edges. The yellow ellipses, blue ellipses, and red ellipses represent the lncRNAs, miRs, and genes, respectively. *Note*: Red lines indicate a negative correlation, and black lines indicate a positive correlation.

**Figure 5:**
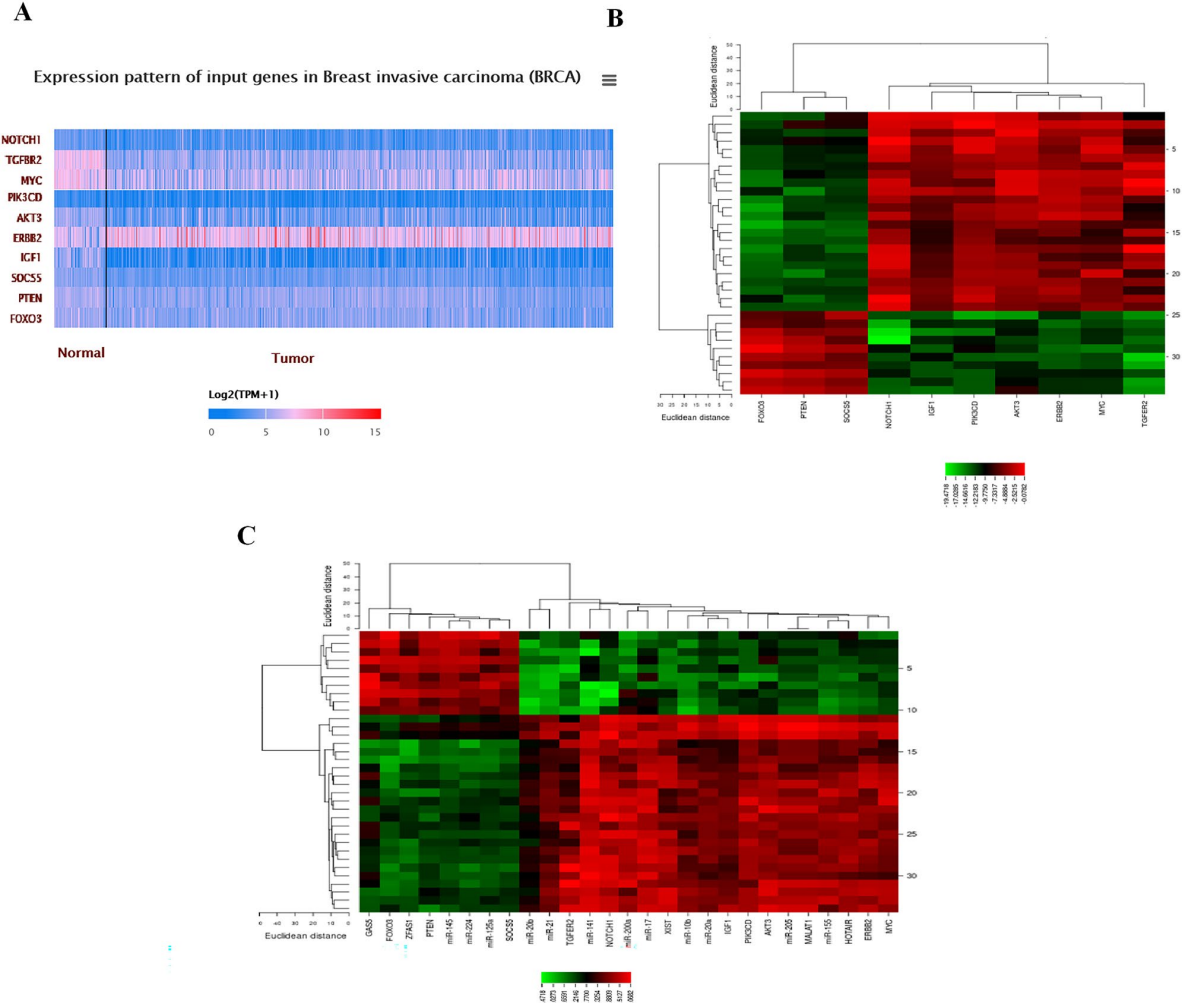
A plot heatmap to show the gene expression profile of DEGs in both bioinformatics (**A**) and experimental data (−∆CT) (**B**,**C**). The green color indicates down-regulated genes, and the red indicates up-regulated genes between tumor and normal samples.

Our results showed that 5 lncRNAs were involved in regulating 12 miRs. 10 target genes regulated by miRs were then identified^18^. Thus, 5 lncRNAs, 11 miRs, and 10 mRNAs were directly related to the lncRNA-miR-mRNA network in breast cancer (Fig. 4). Moreover, we created a heat map of the expression of the candidate lncRNAs, miRs, and mRNAs using CIMminer (https://discover.nci.nih.gov/ cimminer/home.do)^18^ (Fig. 5).

### The expression levels of TGFβ, IL-17, IFNγ, and IL-β proteins

The expression levels of TGFβ, IL-17, IFNγ, and IL-β proteins were measured using the ELISA technique. TGFβ, IL-17, IFNγ, and IL-β expressions were significantly increased in the patients (pre-treatment) compared to the control group (P < 0.05). Unlike the non-aspirin group, the proteins' expression levels were significantly decreased following aspirin consumption; however, their expressions were significantly lower in aspirin users than in non-aspirin users (Table 7).

**Table 7:** The expression levels of TGFβ, IL-17, IFNγ, and IL-β proteins by ELISA assay in response to aspirin consumption in patients with breast cancer.

Indexes	Groups				
	Control	ASA−		ASA+	
		Pre-treat	Post-treat	Pre-treat	Post-treat
TGFβ (pg/ml)	56.5 ± 2.5	*347 ± 25	332 ± 10	*350 ± 9.5	^#,$^ 124 ± 7.0
IL-17 (ng/ml)	5.6 ± 3.5	*99 ± 7.5	89 ± 6.5	*96 ± 9.0	^#,$^ 7.5 ± 9.0
IFNγ (pg/ml)	5.3 ± 2.5	*28 ± 6.8	25 ± 4.5	*29 ± 4.5	^#,$^ 7.5 ± 3.5
IL-β (pg/ml)	16.8 ± 5.5	*447 ± 30	432 ± 49	*455 ± 45	^#,$^ 18.0 ± 6.4

ASA−: Non-aspirin group, ASA+: Aspirin group.

*P < 0.05 compared to the control group.

^#^P < 0.05 compared to Pre-treat ASA+.

^$^P < 0.05 compared to Post-treat ASA−.

### The expression of onco-lncRNAs, oncomiRs, and oncogenes

**Figure 6:**
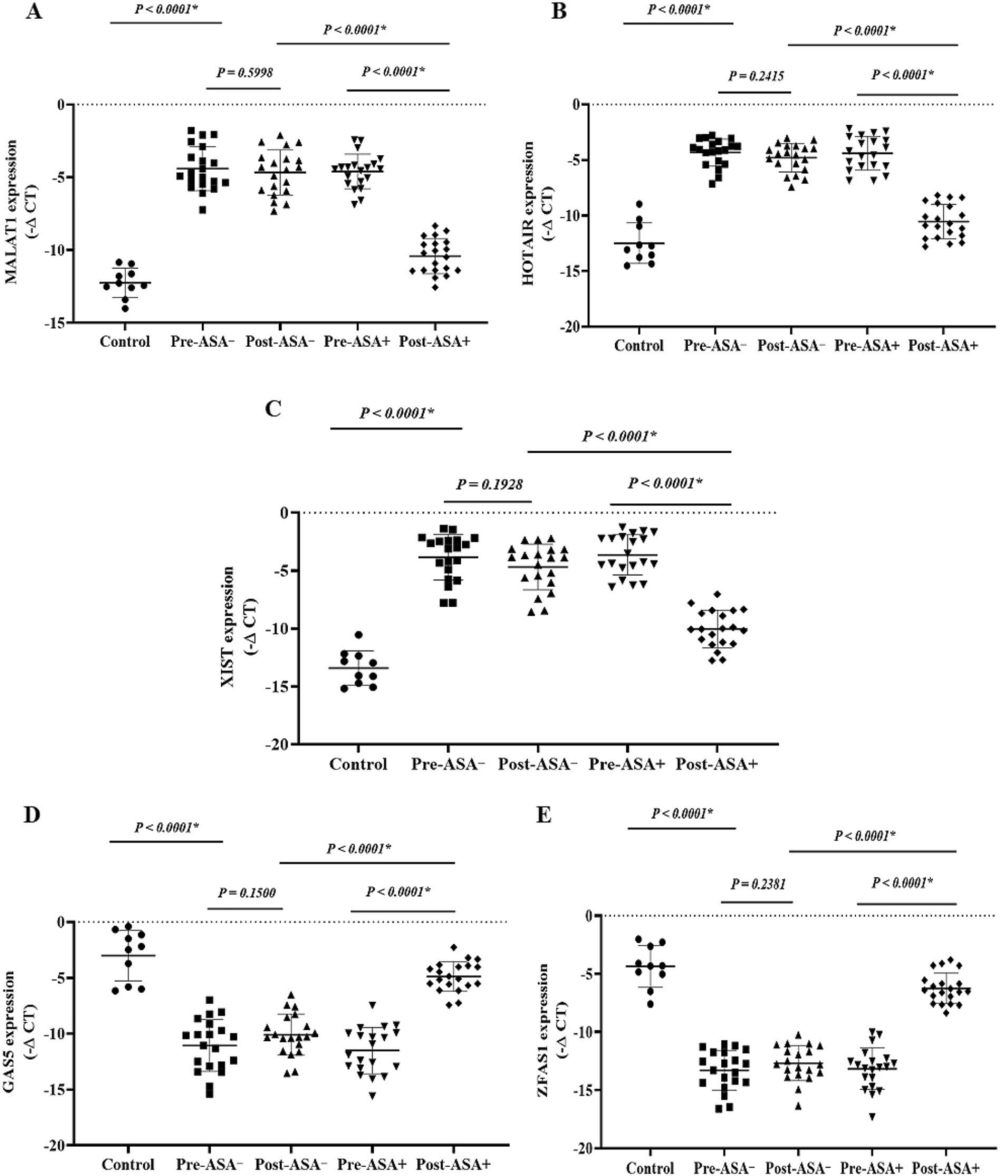
The relative expression of the candidate lncRNAs in the breast cancer patients. The relative expression levels of the lncRNAs were normalized by a reference RNA. The oncolncRNAs included: (**A**) MALAT1, (**B**) HOTAIR, and (**C**) XIST. Tumor suppressor lncRNAs included: (**D**) GAS5 and (**E**) ZFAS1. ASA−: Non-aspirin group, ASA+: Aspirin group. The expression levels of the lncRNAs were calculated using the –ΔCT method.

**Figure 7:**
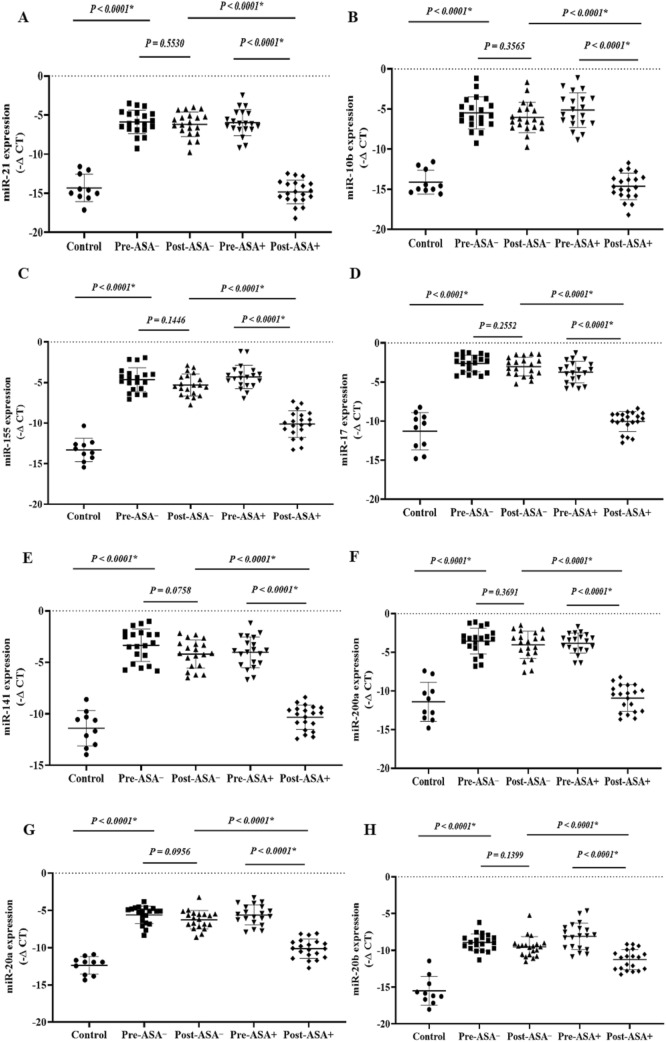

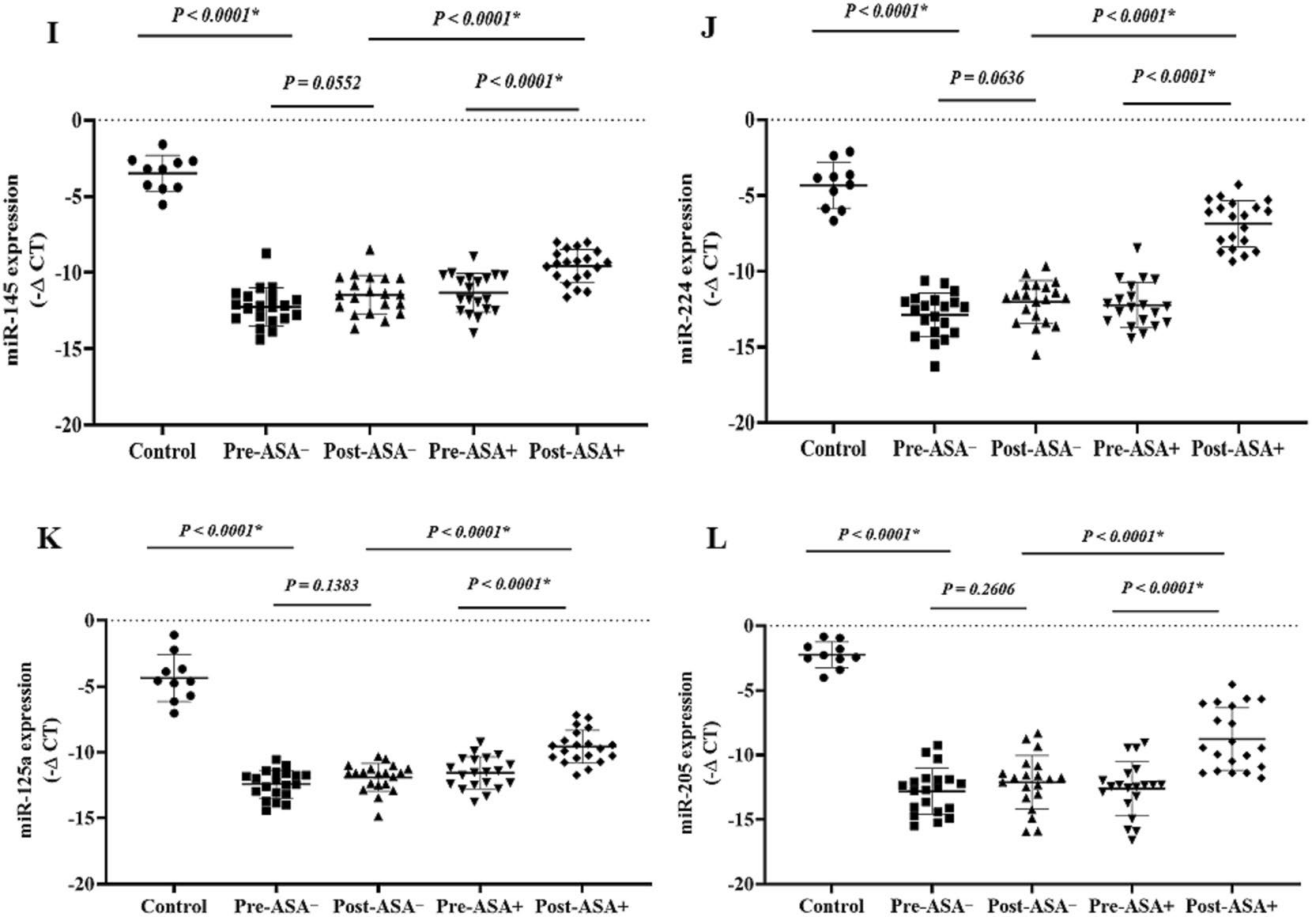
The relative expression of the candidate miRs in the breast cancer patients. The relative expression levels of the miRs were normalized by a reference RNA. The oncomiRs included: (**A**) miR-21, (**B**) miR-10b, (**C**) miR-155, (**D**) miR-17, (**E**) miR-141, (**F**) miR-200a, (**G**) miR-20a, and (**H**) miR-20b. Tumor suppressor miRs included: (**I**) miR-145, (**J**) miR-224, (**K**) miR-125a, and (**L**) miR-205. ASA−: Non-aspirin group, ASA+: Aspirin group. The expression levels of the miRs were calculated using the –ΔCT method.

**Figure 8:**
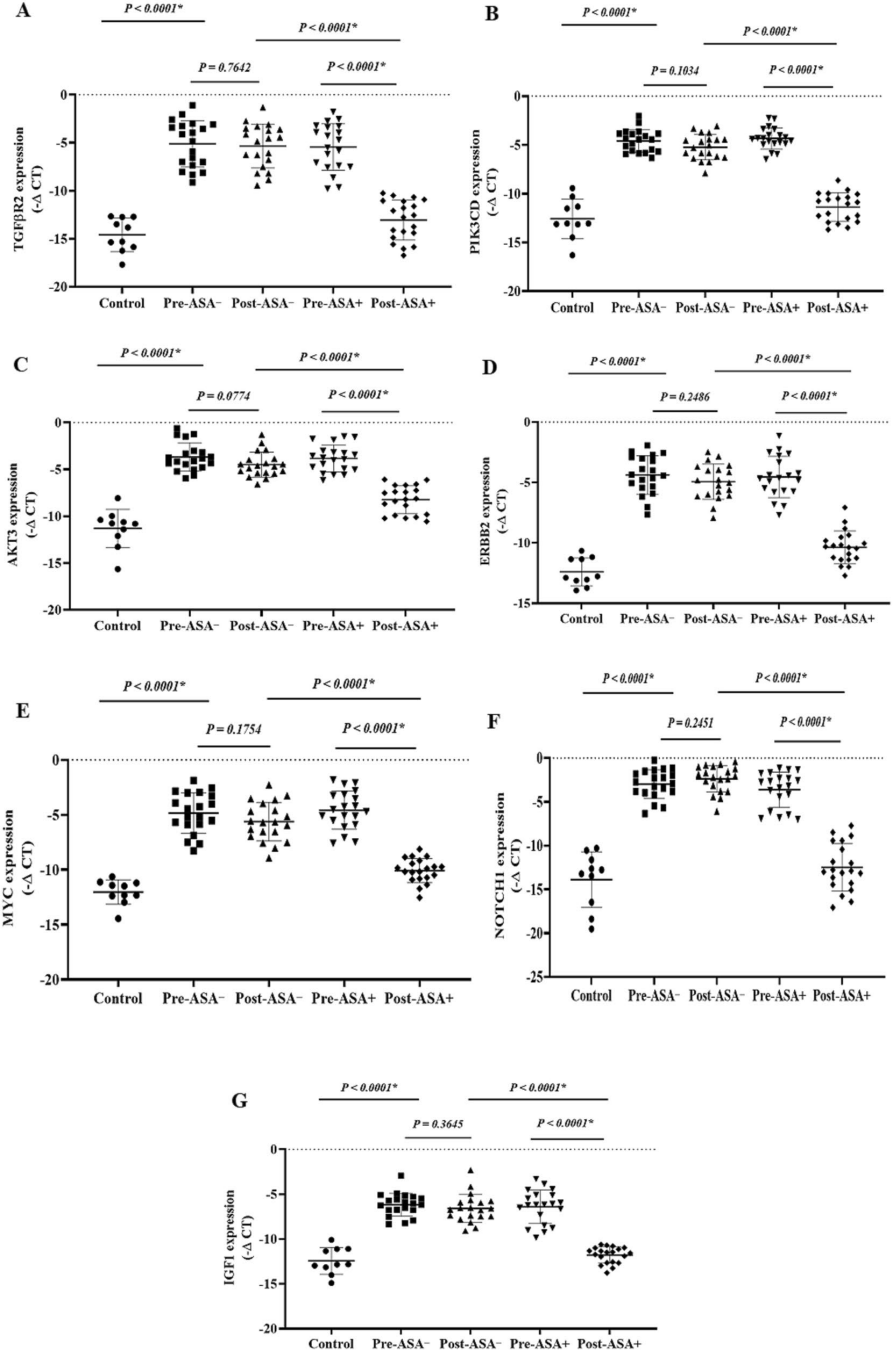

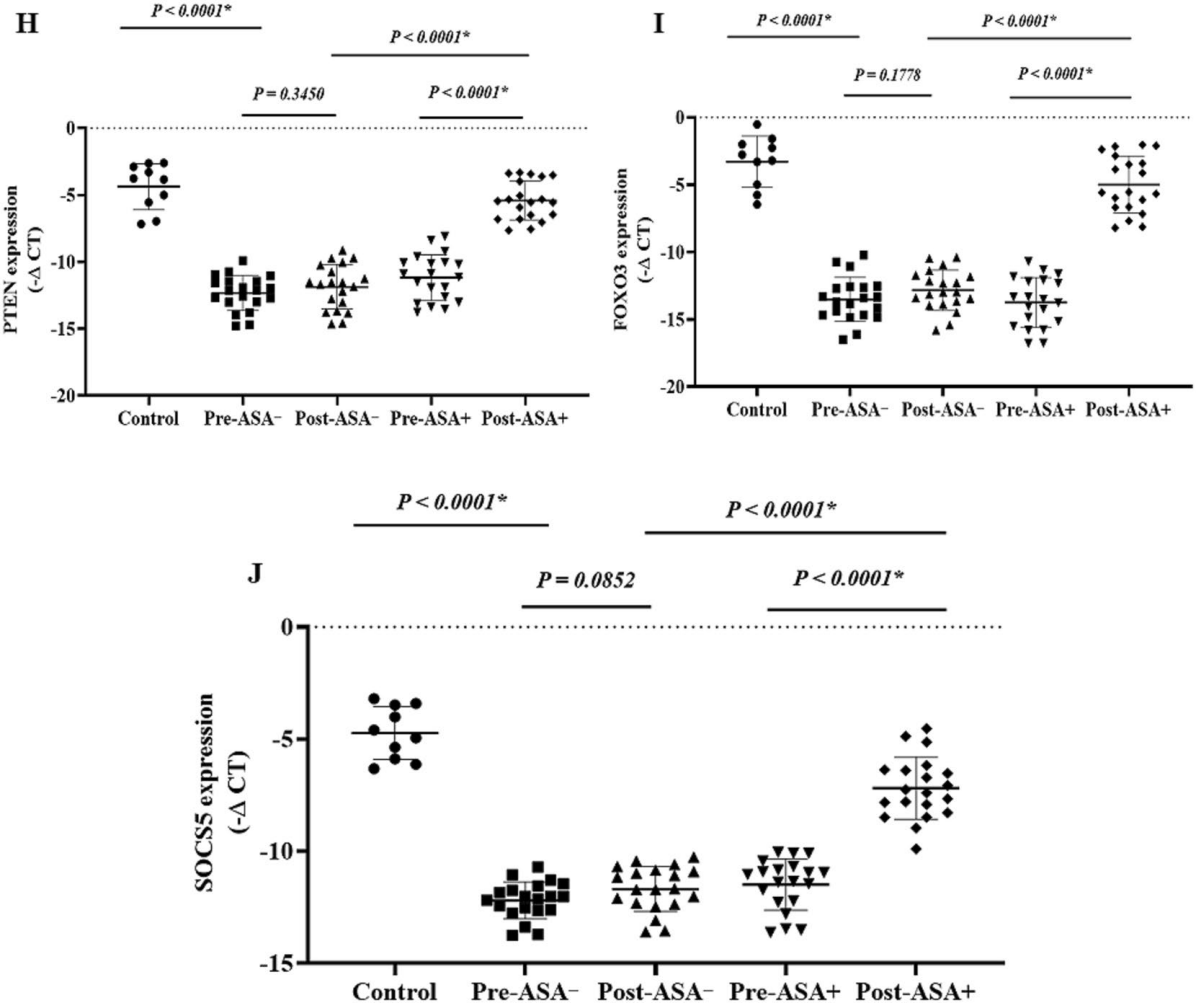
The relative expression of the candidate mRNAs in the breast cancer patients. The relative expression levels of the genes were normalized by a reference gene. The oncogenes included: (**A**) TGFβR2, (**B**) PIK3CD, (**C**) AKT3, (**D**) ERBB2, (**E**) MYC, (**F**) NOTCH1, and (**G**) IGF1. Tumor suppressor genes included: (**H**) PTEN, (**I**) FOXO3, and (**J**) SOCS5. ASA−: Non-aspirin group, ASA+: Aspirin group. The expression levels of the mRNAs were calculated using the –ΔCT method.

The expression levels of the onco-lncRNAs (Fig. 6A–C), -miRs (Fig. 7A–H), and -mRNAs (Fig. 8A–G) were significantly increased in the patients (pre-treatment) compared to the control group (P < 0.05). Unlike the non-aspirin group, the expression levels of the onco-lncRNAs, -miRs, and -mRNAs were significantly decreased following aspirin consumption (P < 0.0001).

### The expression of the tumor suppressor lncRNAs, miRs, and mRNAs

The expression levels of the tumor suppressor -lncRNAs (Fig. 6D,E), -miRs (Fig. 7I–L), and -mRNAs (Fig. 8H–J) were significantly decreased in the patients (pre-treatment) compared to the control group (P < 0.05). The tumor suppressor -lncRNAs, -miRs, and -mRNAs were significantly higher in aspirin users than non-aspirin users (P < 0.0001).

### The candidate miRs and mRNAs as predictive targets

**Figure 9:**
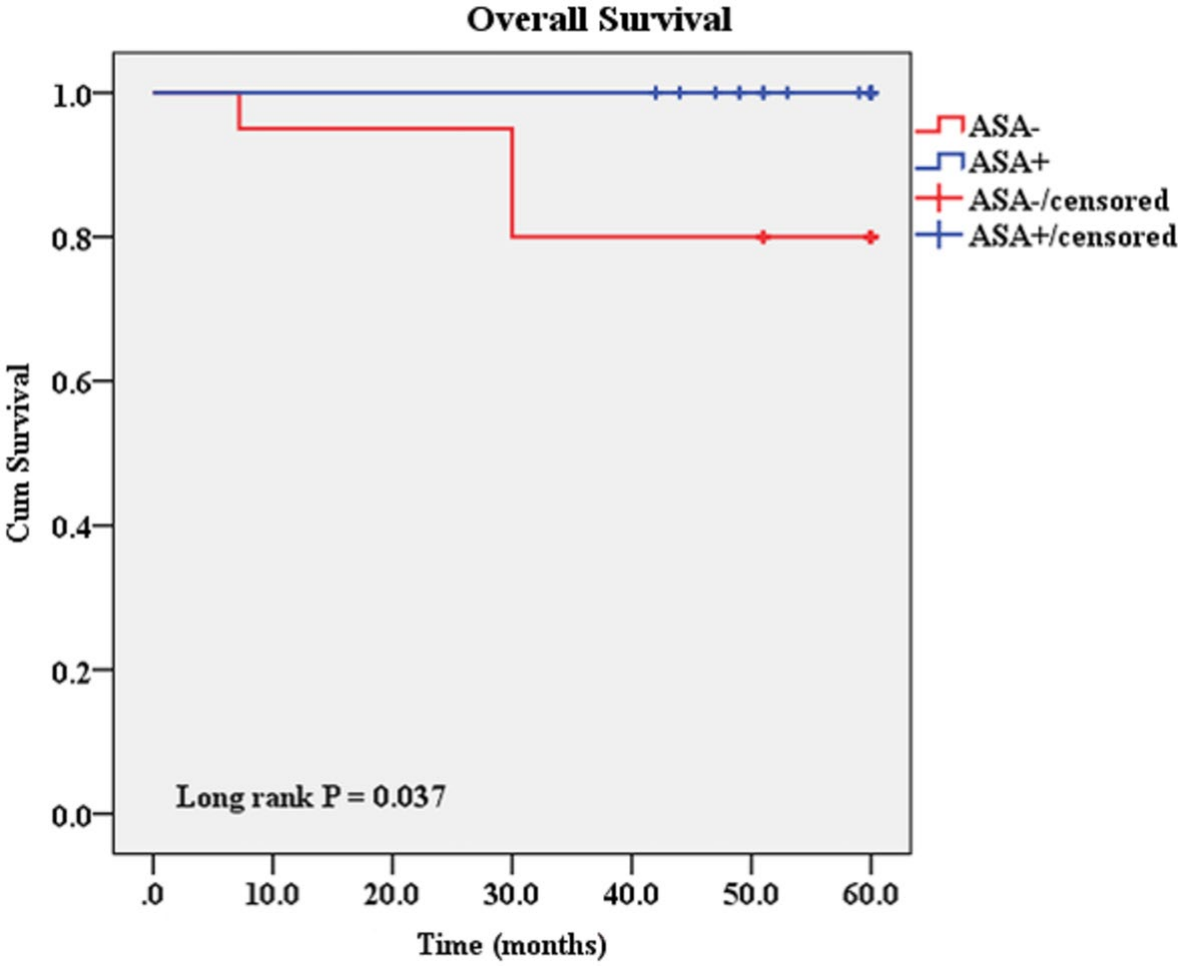
A Kaplan–Meier analysis of 5-year overall survival between the Aspirin and non-aspirin groups. A longer overall survival rate was seen after aspirin consumption.

Our results have exhibited a longer overall survival rate after aspirin consumption than the non-aspirin group (Fig. 9). The mean follow-up duration of the patients was 4.7 ± 1.3 years. Four patients were deceased due to breast cancer disease in the non-aspirin group despite the Aspirin group. In addition, one patient had a recurrence and underwent a new course of treatment in the non-aspirin group.

## Discussion

In the present study, we measured the effects of aspirin consumption on the expression profiles of lncRNAs, miRs, and mRNAs in patients with non-metastatic early luminal A breast cancer. Using computational approaches, we first constructed a network of the lncRNA-miR-mRNA based on our multi-level methodology. 5 lncRNAs, 12 miRs, and 10 mRNAs have been shown to have significant differential expressions in cancerous tissues compared to non-cancerous tissues, improved after aspirin consumption.

To our knowledge, this is the first study to demonstrate aspirin's effects on competing endogenous RNA (ceRNA; lncRNA-miRNA-mRNA) perturbation. They can be promising tools for early detection, prognosis, and monitoring treatment effects. Most of the retrieved biomarkers from our computational analyses have been shown to play essential roles in breast cancer. For instance, miR-21 can increase cell growth and metastasis^19^. MiR-20 family members may also contribute to cell proliferation, migration, invasion, and angiogenic reactions^20,21^. Similar to our results, other molecules such as HOTAIR^22^, XIST^23,24^, GAS5^25,26^, Zfas1^27^, miR-125a, miR-155^28^, miR-224^12^, miR-106a^29^, miR-17^30^, miR-141 ^31^, miR-145^32^, miR-200a^33^, miR-196b, miR-193b^34^, miR-196a^35^, miR-205^36^, and miR-342^37^, have been reported to play important roles in breast cancer. At the molecular level, ceRNAs can inhibit protein production by affecting the stability of their target mRNAs^38^. Moreover, a recent meta-analysis showed the converse correlation of the lncRNA levels with the risk of poor outcomes in breast cancer patients ^39^. To our knowledge, the constructed network between these molecules has not been previously reported. It has been established that the cross-talk between lncRNAs and miRs can be a principal component of cancer pathophysiology^40^. In this regard, Zhang et al. (2017) constructed a massive network of lncRNA-miR-mRNA in breast cancer. Accordingly, miR-510 was the most potent miRNA controller and regulator of numerous target genes. Besides, they showed a group of lncRNAs, including PVT1, CCAT1, and linc00861, that interacted with particular clinical biomarkers such as estrogen and progesterone receptors^41^. In this study, we focused on aspirin effects on the constructed network expression. Aspirin could reduce the WNT activity, arresting the cell cycle via the WNT/β-catenin axis. In this setting, Khan et al. (2019) demonstrated that aspirin could inhibit the cell migration and invasion via the down-regulation of WNT/β-catenin, consequently reducing fibromodulin expression^42^. Besides, Tang et al. (2016) used aspirin and ursolic acid to co-treatment against breast cancer. This combination could reduce the metastatic feature of breast cancer via regulating EGFR mediating signaling pathways^43^. It was also shown that aspirin could increase the PI3K pathway inhibitors^44,45^. According to Henry et al. (2017), PI3K pathway inhibitors had limited clinical response despite the high incidence of PIK3CA mutations in breast cancer patients^44^. They showed that aspirin selectively inhibited the growth of mutant PIK3CA breast cells. Besides, it could sensitize mutant PIK3CA cells to PI3K inhibitors. Co-treatment of aspirin and PI3K inhibitors are led to AMPK activation, mTORC1 inhibition, and autophagy induction^44^. Furthermore, Cheng et al. (2018) showed that aspirin-alone treatment reduced the proliferation of estrogen-positive breast cancer cells. Moreover, aspirin could increase the sensitivity of the tamoxifen-resistant breast cancer cells to tamoxifen by inhibiting c-Myc and cyclin D1 proteins^46^. Consistent results have also been reported in other cancers. Xie et al. (2018) showed that aspirin could enhance the sensitivity of hepatocellular carcinoma cells to doxorubicin via modulation of miR-491/ABCG2 expression^47^. Altogether, the findings of this study and those reported in the literature can support the idea of aspirin co-administration with chemotherapy regimens in breast cancer patients. However, these findings should be interpreted cautiously, and large-scale clinical trials should be conducted to assess the co-administration effects of aspirin with different chemotherapy agents in breast cancer patients.

As the well-known aspirin effects are anti-inflammatory, we measured a group of cytokines closely related to our constructed ceRNA network. In this respect, aspirin users had decreased levels of TGFβ, IFNγ, IL-1b, and IL-17. Similar to our findings, Ma et al. (2021) observed that low-dose aspirin administration would reduce the COX2 and TGFβ intensity in breast cancer patients previously irradiated^48^. In the early stages of tumorigenesis, TGFβ1 acts as a tumor suppressor by inhibiting cell proliferation, inducing apoptosis, and suppressing growth factors, cytokine, and chemokine production. TGFβ1 overexpression can impair immune surveillance and promote angiogenesis, tumor invasion, and metastasis. Besides, aspirin can inflate the anti-tumoral effects of IFN-α. From a closer look, aspirin could enhance the IFN-α-induced apoptosis via the JAK1/STAT1 pathway^49^. Moreover, earlier studies revealed that the intra-tumoral levels of IL-17 were increased and correlated with the expansion of breast cancer^50^. Cochaud et al. (2013) demonstrated that recombinant IL-17A could activate the ERK1/2 pathway and thus promote resistance to docetaxel-based chemotherapy in various cell lines. Altogether, the current literature showed the unintended effects of IL-17 on breast cancer progression^49^. Further investigations are needed to validate the impact of aspirin on particular cytokines in breast cancer patients.

### Clinical applications

Aspirin is one of the most widely used NSAIDs globally; remarkably, it is still one of the most attractive medicines globally, with an extent of use much beyond its primary usage in controlling fever, pain, and inflammation^51^. For example, the co-prescription of the drugs, such as clopidogrel and oral anticoagulation, with aspirin in populations with coronary artery diseases could reduce the risk of death and myocardial infarction^52^. Moreover, a low dose of aspirin was recommended for pregnant women at high risk of preeclampsia and obstetric antiphospholipid syndrome to prevent diverse pathologies of gestation^53^. Nonetheless, aspirin administration for breast cancer prevention or its treatment is still debatable. So far, plenty of clinical trials have explored the effects of aspirin in breast cancer patients^54^. A recent updated meta-analysis of 38 observational studies yielded that aspirin could reduce the risk of breast cancer patients, such as postmenopausal, hormone receptor-positive tumors, or in situ tumors^55^. Although pooled observational studies have shown that long-term aspirin usage is associated with a low risk of breast cancer incidence, a recent meta-analysis of clinical trials showed that aspirin did not necessarily reduce cancer risk (RR = 1.01, 95% CI: 0.97–1.04). The discrepancies between the clinical and observational studies may reduce the potential clinical practicality of aspirin in breast cancer management. Notwithstanding this dispute, recent clinical trials where aspirin was used as adjuvant therapy or as an add-on strategy showed promising results. According to Joharatnam-Hogan et al. (2019), the regular use of aspirin after standard treatments could prevent recurrence and prolong survival in breast cancer patients^56^. Our results showed that the aspirin users had better expressional biomarkers than the non-aspirin users. Although both groups showed significant improvements in their expressional profiles, these changes were more prominent in aspirin users. These findings suggest that aspirin can increase the efficacy of current chemotherapies by increasing the sensitivity of the cancer cells to chemotherapy.

Interestingly, we could demonstrate this benefit of aspirin in the clinical outcomes such as 5-year overall survival. We followed our patients for a long time and showed that the patients exhibited a longer overall survival rate after aspirin consumption than the non-aspirin group. Similar to our findings, Liue et al. (2021) showed that aspirin reduced breast-cancer-specific death by 31%, and the risk of recurrence/metastasis decreased by 9%. In this respect, aspirin may improve all-cause mortality, specific mortality, and risk of recurrence/metastasis in patients with breast cancer^57^. Sendur et al. (2014) also showed that despite the contradictory results regarding aspirin and breast cancer incidence, its use in breast cancer was associated with improved disease-free survival. Aspirin users had a significantly lower incidence of histological grade II-III tumors, but no effect was found on other clinicopathological properties^58^.

## Conclusion

We demonstrated that adding aspirin to the treatment of breast cancer patients could reduce the expression of oncolncRNAs, oncomiRs, and oncogenes and simultaneously increase the levels of tumor-suppressor lncRNAs, miRs, and mRNAs.

## Methods

### Breast cancer datasets

**Figure 10:**
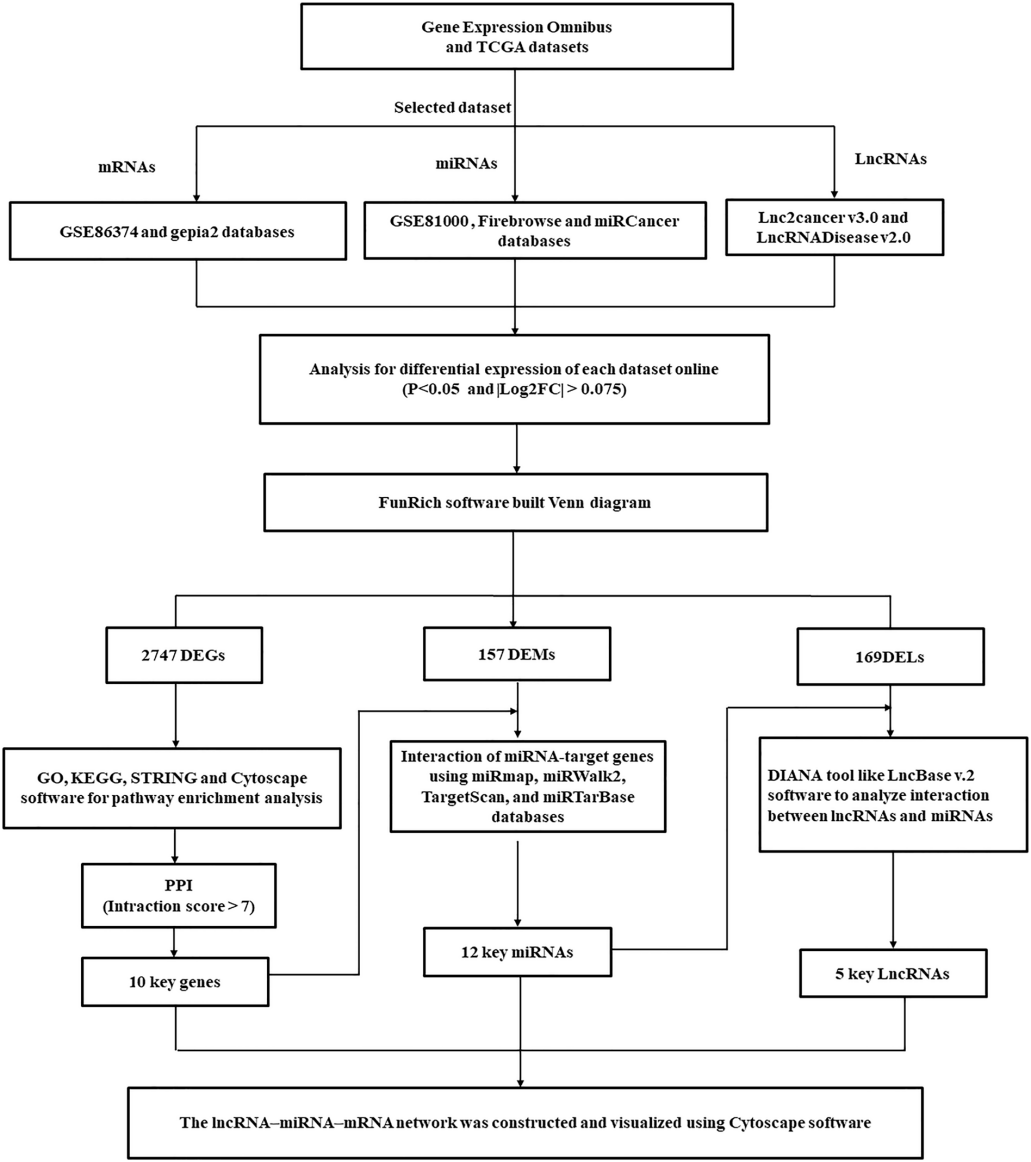
A flowchart diagram for the bioinformatics analysis in the present study.

The expression profiles of miRs (GSE81000) and mRNAs (GSE86374) of luminal A breast cancer patients were downloaded from the Gene Expression Omnibus (GEO) database (https://www.ncbi.nlm.nih.gov/geo/) and analyzed by the GEO2R tools^18,59^. Their differential expression between tumor and standard samples was collected according to the following parameters: with ǀlog2FCǀ > 0.075 and P-value < 0.05. Moreover, the GEPIA2 (http://gepia2.cancer-pku.cn), the cBioPortal (https://www.cbioportal.org), and the Broad Institute's FireBrowse (http://firebrowse.org) are websites for analyzing the DEGs from the TCGA and Genotype-Tissue Expression projects^18,59^. The platforms for miRs of the TCGA dataset included the OncomiR (http://www.oncomir.umn.edu/omcd/), miRGator 3.0 (https://tools4mirs.org), and miRCancerdb (http://mircancer.ecu.edu) databases^60^. The databases for lncRNAs of the TCGA dataset included LncRNADisease (http://www.rnanut.net/lncrnadisease), Lnc2Cancer v3.0 (http://bio-bigdata.hrbmu.edu.cn/lnc2cancer), and TANRIC datasets^61^. Figure 10 shows a flowchart diagram for used bioinformatics analysis.

### The analysis of GO term pathways by the FunRich software

The pathway enrichment analyses of the GO database were executed through the FunRich (http://www.funrich.org) software^18^. Likewise, the same genes were explored for pathway enrichment using the g: Profiler tool (http://biit.cs.ut.ee/gprofiler)^59^. At last, the miR/target gene regulatory network was built using the Cytoscape (https://cytoscape.org) software^59^.

### LncRNA–miR–mRNA network construction

The lncRNA–miR–mRNA network was constructed and visualized using Cytoscape software based on the ceRNA theory^59^. Here, the nodes and edges represent extensive biological data described previously^18^. A network analysis was performed using a Cytoscape plug-in to explore the structure and feature of the lncRNA–miR–mRNA competing triplets^59^.

### Correlation analysis among lncRNAs, miRs, and mRNAs

The correlation coefficient was calculated among lncRNAs, miRs, and mRNAs. The absolute value of the correlation coefficient equal to or more than 0.5 represented a significant correlation^18^.

### Sample collection

This study is part of an ongoing randomized clinical trial registered in the Iranian randomized control trial (IRCT2016080818745N11). All participants were informed of the current research objectives, study protocol, and informed consent to participate in the study. The proposal was approved by the Ethics Committee of Tehran University of Medical Sciences and followed the Helsinki Declaration's ethical principles. Forty patients with luminal A breast cancer referred to the Cancer Institute and Arash Women's Hospitals (two centers affiliated with Tehran University of Medical Sciences, Tehran, Iran) were entered into the study between April 2016 and March 2018^18^. Besides, ten normal-risk women who had attended the breast clinic for screening purposes and had healthy breasts were entered as the controls. The aim and protocol of the study were explained to all participants, and they all provided written informed consent. The right to withdraw from the survey was reserved for all patients at any time.

In all patients with breast cancer, 10 cc of blood was withdrawn twice at a three-month interval at the point of entry and at the end of the study period for each participant (defined below). The blood was centrifuged at 3000 g for 5 min, and the plasma was preserved at − 80 °C. The patients' characteristics included age, tumor size, nodal status, histologic type, Her2, Ki-67, and hormone receptor status^62^.

### Inclusion criteria for patients with breast cancer

1. Desire to participate
2. Age 20–70 years
3. Invasive ductal carcinoma of the breast
4. Luminal A breast cancer (ER+, PR+, Her2^−^, and ki-67 < 15%)
5. Early breast cancer confined to the breast
6. Tumor size larger than 10 mm
7. Undergoing adjuvant chemotherapy

### Inclusion criteria for healthy women

1. Female
2. Desire to participate
3. Age 20−70 years
4. No family history of breast cancer in first and second-degree relatives
5. No history of breast cancer
6. No history of benign breast lump
7. Normal breast exam
8. Normal mammography for those 40 years of age or above

### Exclusion criteria for patients with breast cancer

- Regional lymph node involvement
- Evidence of distant metastasis
- Pregnancy or breastfeeding
- Prior long-term aspirin use
- History of sensitivity to aspirin
- Platelet count < 100,000/µL
- History of coagulopathy or use of anti-coagulative agents
- History of other cancers, peptic or duodenal ulcers, diabetes, hypertension, acquired immunodeficiency syndrome (AIDS), liver and cardiovascular diseases

### Exclusion criteria for healthy women

- Pregnancy or breastfeeding
- Prior long-term aspirin use
- Platelet count < 100,000/µL
- History of coagulopathy or use of anti-coagulative agents
- History of other cancers, diabetes, hypertension, AIDS, liver and cardiovascular diseases

### Randomization, allocation, and blinding

Eligible patients with breast cancer were randomly divided into the Aspirin and non-aspirin groups. Randomization was performed according to a table of random numbers. The allocation of treatments was performed in a 1:1 ratio, and the treatments were assigned using a sealed envelope. The oncologist in charge of the chemotherapy and the patients themselves knew about the groupings (except for the surgeons responsible for the patient and the researchers who collected the blood samples and performed the molecular and cellular tests) were blind to it. According to histologic results of the surgical specimens after the operation and the oncologist's decision, several patients did not need chemotherapy and only underwent endocrine therapy as their systemic adjuvant treatment; these patients were withdrawn from the study. The other patients received their chemotherapy regimen of adriamycin, cyclophosphamide, and a taxane.

### Interventions

**Figure 11:**
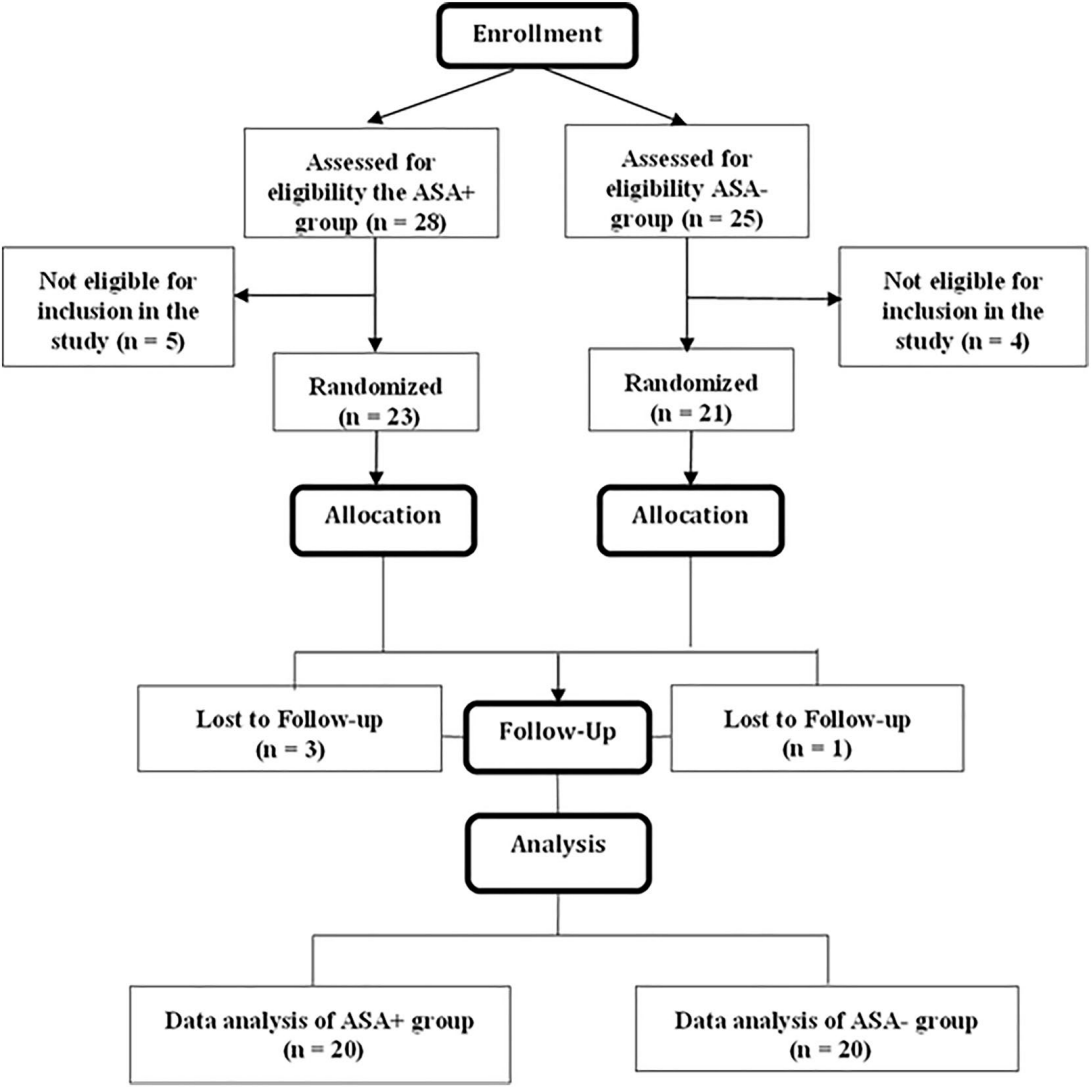
A flowchart of the present trial strategy. ASA−: Non-aspirin group, ASA+: Aspirin group.

The patients in the Aspirin group received an oral daily dose of 80 mg over three months (Fig. 11). Aspirin administration was initiated after the operation during chemotherapy and continued for three months throughout chemotherapy in all patients. The non-aspirin group received no aspirin or other NSAID during the first three months of the chemotherapy.

### Primary outcomes

We measured the expression of the lncRNAs (Table 5), miRs (Table 1), and mRNAs (Table 4) as the primary outcomes before (baseline) and after three months of the intervention in the Aspirin and non-aspirin groups.

### Secondary outcomes

We evaluated the protein levels of TGFβ, IFNγ, IL-17, and IL-1β pre-and-post intervention (Table 7) as the secondary outcomes before (baseline) and after three months of the intervention in the Aspirin and non-aspirin groups.

### Evaluation of TGFβ, IFNγ, IL-17, and IL-1β proteins

ELISA test was used to evaluate the protein levels of TGFβ, IFNγ, IL-17, and IL-1β. The samples were first lysed using a lysis buffer. The protein levels of anti-TGFβ (ab193715, Sensitivity: 1.5 pg/ml, Range: 1.5–500 pg/ml), anti-IFNγ (ab174443, Sensitivity: 470 pg/ml, Range: 0.468–30 ng/ml), anti-IL-17 (ab119535, Sensitivity: 0.5 pg/ml, Range: 1.6–100 pg/ml), and anti-IL-1β (ab46052, Sensitivity: 6.5 pg/ml, Range: 15.6–500 pg/ml) were determined by a sandwich ELISA as follows: aliquots of 100 μl/well (5–10 μg/ml; monoclonal antibody) of anti-TGFβ, anti-IFNγ, anti-IL-17, and anti-IL-1β were used to coat 96-well plates and incubated overnight at 4 °C. Plates were blocked with PBS containing 1% bovine serum albumin (BSA) for one hour at room temperature, followed by washing with washing buffer (PBS) containing 0.1% BSA plus 0.05% Tween 20. Supernatants were diluted at 1:4 or 1:2 with PBS and dispensed into the wells. To generate a standard curve, TGFβ, IFNγ, IL-17, and IL-1β, were used at a concentration range of 25 ng/ml diluted to 100 pg/ml in 10 serial dilutions in PBS plus 1% BSA. After 2-h incubation at room temperature, plates were washed, and 100 μl of 0.5 μg/ml of biotin anti-TGFβ, anti-IFNγ, anti-IL-17, and anti-IL-1β antibodies were added to each well. After two-hour incubation at room temperature, plates were thoroughly washed, and 100 μl of a 1:10,000 dilution of peroxidase-streptavidin conjugate were added to each well. Plates were incubated at room temperature for one hour. After washing off the unbound antibody, 100 μl of TMB-peroxidase substrate/chromogen solution was added to each well and incubated for 10–20 min. The reaction was stopped with 100 μl of 1 M H_3_PO_4_. An automated ELISA reader determined absorbance at 450 nm.

### Real-time PCR analysis

The RNA was extracted from the plasma samples. Plasma (250 μl) was added to 750 μl TRIzol (Beijing Tiangen Biotech Co., Ltd.). RNA extraction was then carried out according to the manufacturer's instructions. The absorbance ratio (A260/280) of total RNA, between 1.8 and 2.2, was determined using an ultraviolet (UV) spectrophotometer. According to the manufacturer's recommendations, the miRcute miRNA cDNA First-Strand Synthesis kit (Beijing Tiangen Biotech Co., Ltd.) for miRs quantification and the cDNA Synthesis Kit Manual (TAKARA BIO INC. Cat. 6 30 v.0708) for mRNAs and lncRNAs quantification were used. Then, cDNA was used in each real-time PCR assay with the miRcute miR Fluorescence Quantitative Detection kit (Tiangen Biotech Co., Ltd.) for miRs. The cycling conditions were the pre-denaturation at 94 °C for 2 min, followed by 40 cycles of 94 °C for 20 s and 60 °C for 34 s. The SYBR Green method (AccuPower Green Star qPCR Master Mix; Bioneer, Korea) was used for genes and lncRNAs. PCR cycling was performed as follows: one cycle at 95 °C for 10 min, 40 cycles at 95 °C for 20 s, and 60 °C for 45 s. The melting curve analysis was run from 60 to 95 °C to confirm specific amplification^18,59^. The expression of U6 and B-actin was used to normalize miRs, lncRNAs, and genes as the Internal Reference Gene. The list of primers has shown in Table 8. The qRT-PCR reactions were performed using an ABI StepOne plus System (Applied Biosystems; Thermo Fisher Scientific, Inc.). The expression level of the genes was calculated using the − ΔCT method. ΔCT was calculated by subtracting the CT values of U6 and B-actin from the targets^63,64^.

**Table 8:** The list of primers for real-time PCR.

Genes/miRNAs	Forward primer	Reverse primer
TGFBR2	GCTTTGCTGAGGTCTATAAGGC	GGTACTCCTGTAGGTTGCCCT
PIK3CD	TGGCGGATAGACATACATTGC	ACCAGTAGGCAACCGTGAAG
AKT3	TGAAGTGGCACACACTCTAACT	CCGCTCTCTCGACAAATGGA
ERBB2	CAGGGGTGGTATTGTTCAGC	GGGAAACCTGGAACTCACCT
SOCS5	TGAGCCTACCACACGGTATTATG	GATTGTACTTACTCAATGACCT
IGF1	GCTCTTCAGTTCGTGTGTGGA	GCCTCCTTAGATCACAGCTCC
MYC	GACCAGAAAAGTAGCTGCCG	GCCCGGATGTGCACTAAAAT
NOTCH1	ACAGTCTGGGCCTATGAAACC	TGTGAACGTGATGTCAACGAG
PTEN	GGTGGGTTATGGTCTTCAAAAGG	TGGATTCGACTTAGACTTGACCT
FOXO3	CACGGCTTGCTTACTGAAGG	TCACGCACCAATTCTAACGC
B-actin	CACCATTGGCAATGAGCGGTTC	AGGTCTTTGCGGATGTCCACGT
miR-17	GCCAGAAGGAGCACTTAGGGCA	TGGTGACAGCTGCCTCGGGA
miR-200a	GGCTGGGGACCTGAGGCGAT	CGGGGGCCCTCGTCTTACCC
miR-205	CCTCCATCCTTCATTCCACCG	GTTTCCGTCGTTCTAATGCGAA
miR-141	CCCCCATCCAGAGGGGTGAAGG	GGCTCCCGGGTGGGTTCTCT
miR-21	CGCCATGTAAAGTGCTTATAGTGC	CGATTCATTTGTTAGCGAGCGG
miR-10b	TTGGAGTTACCCTGTAGAACCG	TAAGCACGAGACTTACGGAGGA
miR-125a	GTTGATTCTCCCTGAGACCCTTTA	GTCCTCACAACGATTCCACAAG
miR-155	CGCCATGTTTAATGCTAATCGTGA	TTCCAGAAACCGATCAGAGTGT
miR-20a	CGCCATGTAAAGTGCTTATAGTGC	CGATTCATTTGTTAGCGAGCGG
miR-20b	GCCCTAAATGCCCCTTCTGGCA	ACACTGCACAGTCCCCACCATCT
miR-224	CGTTTGCCAAGTCACTAGTGGT	TTGTAAGCACGCTACATCCTGA
miR-145	GTAGGAGGTCCAGTTTTCCCAG	TGAACTTCGCAACTACCGTTTG
U6	ATGCAGTCGAGTTTCCCACAT	CCATGATCACGAAGGTGGTTT
MALAT1	GACTTCAGGTCTGTCTGTTCT	CAACAATCACTACTCCAAGC
XIST	CTCCAGATAGCTGGCAACC	AGCTCCTCGGACAGCTGTAA
GAS5	CTTCTGGGCTCAAGTGATCCT	TTGTGCCATGAGACTCCATCAG
HOTAIR	GCTTCTAAATCCGTT	CTCCACGGTAAATCCGGCAG
ZFAS1	AACCAGGCTTTGATTGAACC	ATTCCATCGCCAGTTTCT

### Clinical outcomes

We evaluated the association between the intervention groups with the clinicopathological feature of patients, such as the 5-year overall survival.

### Data analysis

The sample size was calculated based on a study by Chen et al. (2016) and the differences in miR-21 expression in healthy subjects and patients with breast cancer^65^. The sample size was 23 in each arm, considering the alpha error less than 0.05 (α) and the research power of 95% (1-β). The data analyses were performed by GraphPad Prism 7.0 (https://www.graphpad.com). We used the t-test and the Mann–Whitney to analyze the parametric and non-parametric data in two groups. The 5-year overall survival rate was evaluated using the Kaplan–Meier method. All data were presented as mean ± SD. P-value < 0.05 was considered to be statistically significant.

### Ethical approval

The experimental procedures and care protocols were approved by a review board committee of Tehran University of Medical Sciences (No: IR.TUMS.VCR.REC.1397.606) and registered by the Iranian Randomized Control Trial (IRCT) ethical board (No: IRCT2016080818745N11). Written informed consent was obtained from each participant before the sample collection.
